# Heart rate variability and baroreceptor reflex sensitivity in early- versus late-onset preeclampsia

**DOI:** 10.1371/journal.pone.0186521

**Published:** 2017-10-20

**Authors:** Thomas Michael Weber, Helmut Karl Lackner, Andreas Roessler, Ilona Papousek, Vassiliki Kolovetsiou-Kreiner, Miha Lucovnik, Karin Schmid-Zalaudek, Uwe Lang, Manfred Georg Moertl

**Affiliations:** 1 Department of Physiology, Medical University of Graz, Graz, Austria; 2 Department of Medical Engineering, Graz University of Technology, Graz, Austria; 3 Department of Psychology, Biological Psychology Unit, University of Graz, Graz, Austria; 4 Department of Obstetrics and Gynecology, Medical University of Graz, Graz, Austria; 5 Department of Perinatology, Division of Obstetrics and Gynecology, University Medical Centre Ljubljana, Ljubljana, Slovenia; 6 Department of Obstetrics and Gynecology, Clinical Center, Klagenfurt, Austria; University of Washington, UNITED STATES

## Abstract

**Objective:**

To determine whether there are differences in autonomic nervous system function in early- versus late-onset preeclampsia.

**Methods:**

Matched case-control study. Cases were defined as singleton pregnancies with preeclampsia at < 34^+0^ weeks of gestation (early-onset preeclampsia) and ≥ 34^+0^ weeks of gestation (late-onset preeclampsia). For each case in each of the preeclampsia subgroups, three „control”uncomplicated singleton pregnancies were matched by maternal age, height, and week of gestation. Blood pressure and heart rate were measured continuously for 30 minutes in each participant. Baroreceptor reflex sensitivity (assessed using sequence technique), time and frequency domain heart rate variability measures, as SDNN, RMSSD, LF_RRI_, HF_RRI_ and LF/HF_RRI_ of R-R intervals, were compared between groups (p<0.05 significant).

**Results:**

24 women with preeclampsia (10 with early-onset and 14 with late-onset preeclampsia) and 72 controls were included in the study. SDNN, RMSSD and HF_RRI_ were significantly higher in the late-onset preeclampsia group compared to gestational age matched controls (p = 0.033, p = 0.002 and p = 0.018, respectively). No significant differences in SDNN RMSSD and HF_RRI_ between early-onset preeclampsia group and gestational age matched controls were observed (p = 0.304, p = 0.325 and p = 0.824, respectively). Similarly, baroreceptor reflex sensitivity was higher in late-onset preeclampsia compared to controls at ≥ 34 weeks (p = 0.037), but not different between early-onset preeclampsia compared to controls at < 34 weeks (p = 0.50).

**Conclusions:**

Heart rate variability and baroreceptor reflex sensitivity are increased in late- but not early-onset preeclampsia compared to healthy pregnancies. This indicates a better autonomic nervous system mediated adaptation to preeclampsia related cardiovascular changes in late-onset disease.

## Introduction

Preeclampsia, a multisystem disorder characterized by new-onset hypertension and proteinuria or end-organ dysfunction after 20 weeks of gestation, affects 2 to 8% of pregnancies [1–7]. It is associated with high risk of fetal and neonatal complications, as well as maternal morbidity and mortality [7–10]. The timing of disease onset is an important indicator of disease severity [11–15]. Early-onset preeclampsia (< 34 weeks' gestation) and late-onset preeclampsia (≥ 34 weeks' gestation) share several clinical and etiologic features, but their risk factors differ significantly, and early-onset disease often has a more severe course leading to worse outcomes compared to late-onset disease [11–15]. Knowledge of differences in maternal cardiovascular function in early- vs. late-onset preeclampsia is extremely important in developing individualized management strategies for patients with these two different preeclampsia subtypes.

The autonomic nervous system plays a central role in cardiovascular adaptation to pregnancy-related hemodynamic changes [16–18]. Several studies have shown that the increases in peripheral vascular resistance and blood pressure that characterize preeclampsia are mediated, at least in part, by a substantial increase in sympathetic vasoconstrictor activity [19–21]. Our group and others have shown that autonomic nervous system functioning can be non-invasively assessed during pregnancy by analyzing heart rate variability and baroreceptor reflex sensitivity (BRS) [18,22,23]. These measurements could, therefore, be of great clinical value in the prediction, diagnosis and management of preeclampsia. However, it is still not clear whether changes in autonomic nervous system function differ between normal pregnancies and those complicated by early- as well as late-onset preeclampsia.

The aim of the study was to determine whether there are significant differences in autonomic nervous system function in pregnancies complicated by early- compared to late-onset preeclampsia and normal pregnancies.

## Materials and methods

### Study population

The study design utilized a matched case-control methodology. Cases were defined as singleton pregnancies with preeclampsia at < 34^+0^ weeks of gestation at the time of diagnosis (early-onset preeclampsia) and ≥ 34^+0^ weeks of gestation at the time of diagnosis (late-onset preeclampsia; the notation of e.g. 35^+4^ weeks of gestation is equivalent to 35 weeks plus four days) hospitalized at our tertiary perinatal center. For each case in each of the preeclampsia subgroups, three „control”singleton pregnancies without preeclampsia and pre-existing diseases (e.g. insulin-dependent diabetes, cardiovascular or renal diseases, etc.) were matched by maternal age, height, and week of gestation.

Gestational age was derived from the last menstrual period that was confirmed by first trimester ultrasound scans. Preeclampsia was defined using the American College of Obstetricians and Gynecologist Task Force on Hypertension in Pregnancy recommendations [7].

### Heart rate variability and baroreceptor reflex sensitivity assessment

After participants were familiarized with the test protocol, equipment and personnel, electrodes were attached and patients were positioned in the 15° left lateral position, ensuring a continuous venous blood flow to the heart. During the whole procedure the participants were asked not to talk or make abrupt movements. The study protocol consisted of an adaptation period of 20 min and 10 min recording at rest. For analysis a five minute epoch commencing at 4 min of rest was used.

Continuous monitoring of blood pressure (sampling rate, sr = 100Hz, BP_range_ = 50–250 mmHg, ±5 mmHg) and hearth rate (R-R intervals derived from 3-lead electrocardiography, sr = 1 kHz, f_cut-off_ = 0.08–150 Hz) were carried out with the Task Force® Monitor (CNSystems, Medizintechnik AG, Graz, Austria) [24]. Continuous blood pressure was derived from the finger using a refined version of the vascular unloading technique and corrected to absolute values with oscillometric blood pressure measurement by the Task Force® Monitor [24].

For the purpose of this study we analyzed time domain and frequency domain measures of heart rate variability. Time domain measures were computed as the standard deviation of normal-to-normal beat (SDNN) and root mean squared successive differences (RMSSD) of R-R intervals. SDNN reflects sympathetic and to some extent vagal tone whereas RMSSD reflects vagal tone only [25].

For frequency domain measures of the variability of R-R intervals (RRI) we used Fast Fourier Transform with a Hanning window for spectral analysis of cardiovascular signals on the blocks of 5 min epochs, after resampling and removing the trend of 2^nd^ order. Low frequency (LF_RRI_) was defined as 0.04–0.15 Hz, high frequency (HF_RRI_) was defined as 0.15–0.40 Hz, according to published recommendations and LF/HF_RRI_, an indicator of the autonomic state was computed [25]. Because of skewed distribution a natural logarithmic transformation was applied (ln(LF_RRI_), ln(HF_RRI_), ln(LF/HF_RRI_)).

The sequence technique was used for the assessment of BRS [18,26]. This technique is based on identifying consecutive cardiac beats in which an increase in systolic blood pressure is accompanied by an increase in heart rate, or in which a decrease in systolic blood pressure is accompanied by a decrease in heart rate. The regression line between the systolic blood pressure and heart rate produces an estimate of BRS. In the present study an equivalent change in heart rate and systolic blood pressure for at least three consecutive cardiac cycles was defined as a regulatory event if the following criteria were fulfilled: heart rate (R-R interval) variations > 4 ms and systolic blood pressure changes > 1 mmHg.

In addition, further data which are not relevant for this research question were analyzed [27].

### Statistical analysis and ethics statement

Data are expressed as means ± standard deviations. ANOVA was used for comparison between the groups with early-onset preeclampsia vs. late-onset preeclampsia vs. controls. For all tests, a two-tailed p value ≤ 0.05 was considered statistically significant. The software used for statistical analysis was IBM SPSS Statistics for Windows Version 21.0 (Armonk, NY: IBM Corp.).

The study was performed in accordance with the 1964 Declaration of Helsinki and was approved by the Ethics Committee of the Medical University of Graz. Written informed consent was obtained from all participants.

## Results

Twenty-for women with preeclampsia (10 with early-onset and 14 with late-onset preeclampsia) and 72 controls were included in the study. In five women the severity of preeclampsia were classified as *mild* (two in the early-onset, three in the late onset group), in the other cases as *severe*.

Demographic data for the 96 study participants are presented in Table 1. Women with preeclampsia had a higher pre-pregnancy body mass index (BMI) (p = 0.05), and delivered earlier than women in the control group (p<0.001).

**Table 1:** Demographic parameters of the study population.

	Preeclampsia			Controls		
	*all* *(n = 24)*	*Early-onset* *(GA < 34*^*+0*^ *weeks) (n = 10)*	*Late-onset* *(GA ≥ 34*^*+0*^ *weeks*) *(n = 14)*	*all* *(n = 72)*	*GA<34*^*+0*^ *weeks (n = 30)*	*GA* ≥34^*+0*^ weeks *(n = 42)*
Gestational age at inclusion	33^+5^ ± 3^+3^	30^+6^ ± 2^+0^	35^+4^ ± 2^+6^	33^+5^ ± 3^+4^	30^+6^ ± 2^+1^	35^+5^ ± 2^+5^
age (years)	30.3 ± 6.3	31.0 ± 5.9	29.9 ± 6.8	31.9 ± 5.0	33.5 ± 5.0	30.8 ± 4.7
weight (kg)	67.3 ± 18.5	65.7 ± 13.4	68.4 ± 21.8	63.2 ± 10.7	63.6 ± 12.4	63.0 ± 9.4
height (cm)	165 ± 6	164 ± 8	166 ± 6	167 ± 5	167 ± 6	167 ± 5
BMI (kg/m^2^)	24.6 ± 6.1 ^a^	24.4 ± 4.6 ^b^	24.7 ± 7.2	22.5 ± 3.6	22.6 ± 3.8	22.5 ± 3.4
Delivery (day of gestation)	244 ± 23 ^a^	221 ± 12 ^b^	260 ± 12 ^b^	274 ± 10	273 ± 11	275 ± 8

BMI body mass index; GA gestational age (e.g. 30^+6^ ± 2^+0^ represents day of gestation 216 ± 14)

^a^ denotes significant differences (p<0.05) between the preeclampsia group and controls (all)

^b^ denotes significant differences (p<0.05) between subgroups of preeclampsia (< 34 and ≥ 34 weeks) and subgroups of controls (< 34 and ≥ 34 weeks)

Table 2 presents comparisons between groups in terms of heart rate and heart rate variability measures as well as BRS.

**Table 2:** Heart rate variability indexes (standard deviation of normal-to-normal beat (SDNN) and root mean squared successive differences of R-R intervals (RMSSD)) and baroreceptor reflex sensitivity (BRS) in preeclampsia groups vs. controls.

	Preeclampsia			Controls		
	*all* *(n = 24)*	*Early-onset* *(GA < 34*^*+0*^ *weeks)* *(n = 10)*	*Late-onset* *(GA ≥ 34*^*+0*^ *weeks)* *(n = 14)*	*all* *(n = 72)*	*GA<34*^*+0*^ *weeks* *(n = 30)*	*GA* ≥*34*^*+0*^ *weeks* *(n = 42)*
Hr (bpm)	81.2 ± 12.2	83.6 ± 10.8	79.4 ± 13.2	83.3 ± 9.4	85.4 ± 10.0	81.8 ± 8.9
SDNN (ms)	41.0 ± 16.1	33.2 ± 10.7	46.5 ± 17.4 ^b^	36.5 ± 11.2	35.2 ± 9.9	37.1 ± 12.2
RMSSD (ms)	26.8 ± 17.3 ^a^	17.7 ± 9.5	33.3 ± 18.9 ^b^	20.5 ± 9.4	19.9 ± 10.3	21.0 ± 8.9
ln (LF_RRI_) (ms^2^)	5.76 ± 0.91	5.28 ± 0.81	6.11 ± 0.83	5.67 ± 0.81	5.58 ± 0.77	5.74 ± 0.83
ln (HF_RRI_) (ms^2^)	5.11 ± 1.19	4.44 ± 1.18	5.58 ± 0.98 ^b^	4.84 ± 0.94	4.81 ± 0.96	4.87 ± 0.93
ln (LF/HF_RRI_) (-)	0.65 ± 0.84	0.84 ± 1.13	0.52 ± 0.57	0.83 ± 0.73	0.77 ± 0.77	0.88 ± 0.70
BRS (ms/mmHg)	11.7 ± 6.4	9.1 ± 4.4	13.6 ± 7.0 ^b^	10.4 ± 4.2	10.3 ± 4.9	10.4 ± 3.8

GA gestational age

^a^ denotes significant differences (p<0.05) between the preeclampsia group and controls (all)

^b^ denotes significant differences (p<0.05) between subgroups of late- onset preeclampsia and ≥ 34 weeks controls

There were no significant differences in the heart rate between preeclamptic patients and healthy controls overall as well as between the preeclampsia groups compared to gestational age matched controls (overall p = 0.370; early-onset p = 0.638; late-onset p = 0.434).

SDNN did not differ between preeclamptic patients and healthy controls overall (p = 0.132), whereas RMSSD was higher in preeclamptic patients (p = 0.027). Furthermore, SDNN and RMSSD were significantly higher in the late-onset preeclampsia group compared to gestational age matched controls (p = 0.033 and p = 0.002, respectively). No significant differences in SDNN and RMSSD between the early-onset preeclampsia group and gestational age matched controls were observed (p = 0.593 and p = 0.549, respectively).

The ANOVAs yielded no significant differences regarding frequency domain measures ln(LF_RRI_), ln(HF_RRI_) and ln(LF/HF_RRI_) between preeclamptic patients and healthy controls overall (p = 0.664, p = 0.271, and p = 0.237, respectively). However, ln(HF_RRI_) was significantly higher in the late-onset preeclampsia group compared to gestational age matched controls (p = 0.018). No difference for this group were seen for ln(LF_RRI_) and ln(LF/HF_RRI_) (p = 0.167 and p = 0.097, respectively). In a line with the time domain measures no significant differences in the frequency domain measures ln(LF_RRI_), ln(HF_RRI_) and ln(LF/HF_RRI_) between early-onset preeclampsia group and gestational age matched controls were observed (p = 0.304, p = 0.325 and p = 0.824, respectively).

Similarly, BRS values did not differ significantly between the preeclampsia group and controls overall (p = 0.244). BRS was higher in late-onset preeclampsia compared to controls at ≥ 34 weeks (p<0.037), but not statistically different between early-onset preeclampsia compared to controls at < 34 weeks (p = 0.500).

## Discussion

Our results show that late-onset preeclampsia, but not early-onset preeclampsia, is associated with increased heart rate variability and BRS compared to matched healthy pregnancies. The sequence technique used for the assessment of BRS provides an index of regulatory activity of the autonomic nervous system on the R-R intervals. The cardiac branch of the baroreflex, which regulates the interaction between blood pressure and R-R intervals, is one relevant source of parasympathetic influences in cardiac autonomic regulation [26, 28]. Hypertension, which is the cardinal symptom of preeclampsia, is usually accompanied by diminished BRS [29]. The observed increased heart rate variability and BRS in late-onset preeclampsia may indicate that, in opposition to most cases of chronic hypertension and early-onset preeclampsia, the relevant regulation mechanism is largely intact in late-onset preeclampsia. This may indicate a greater ability to flexibly respond to additional demands despite high blood pressure levels in late-onset preeclampsia.

Faber et al. showed that heart rate variability and BRS differed between different hypertensive disorders of pregnancy (chronic hypertension, gestational hypertension and preeclampsia), suggesting that different manifestations of hypertension in pregnancy have different pathophysiological, regulatory, and compensatory mechanisms [30]. However, they did not observe a difference in preeclampsia compared to healthy pregnancies whereas Silver et al. found a significant decrease in BRS associated with hypertensive disorders of pregnancy [31].

In our study no difference in BRS and HRV variables except RMSSD were observed between the 24 preeclamptic women and the 72 case-matched healthy controls. However, the separate analysis of early-onset and late-onset preeclamptic women compared to the matched healthy pregnancies showed a difference in BRS and HRV of late-onset compared to healthy pregnancies. Instead, no difference for early-onset compared to healthy pregnancies were observed supporting the findings of Faber et al. Differences between our findings and their study might also be explained by the small number of patients with early-onset preeclampsia in our study. Furthermore, in contrast to our results, previous studies demonstrated a physiological decrease in heart rate variability and BRS throughout normal pregnancy [18,32]. Therefore, further studies are needed to determine whether BRS and possibly heart rate variability are decreased in early-onset preeclampsia, which would indicate an even greater difference in autonomic nervous system function between the two preeclampsia subtypes. Further studies are also needed to define the effects of antihypertensive medications on BRS and heart rate variability measurements in preeclamptic patients.

Despite the relatively small sample size, the study also has several strengths. It is the first study assessing changes in heart rate variability and BRS in early- and late-onset preeclampsia separately. Furthermore, the methods used for the assessment of heart rate variability and BRS are non-invasive and their validity has been verified in animal studies, numerous clinical studies outside pregnancy, as well as in pregnant women [18,33–37].

In conclusion, the results of our study suggest significant differences in autonomic nervous system responses in early- vs. late-onset preeclampsia. This suggests that these two preeclampsia subtypes should be managed as two distinct entities not only from an etiologic and prognostic standpoint, but also in terms of treatment options. Non-invasive measurements of BRS and heart rate variability could be of great clinical importance not only for prediction and diagnosis of preeclampsia, but also for management of patients with already established clinical signs of disease. As objective markers of autonomic nervous system function these measurements could help clinicians individualizing antihypertensive, volume and diuretic therapy which would lower the risks of side effects and additional end-organ damage in patients with preeclampsia.

## Supporting information

S1 Table Dataset. Dataset of the reported data plus blood pressure values.
(XLS)
